# Antioxidant and Inflammatory Effects of *Nelumbo nucifera* Gaertn. Leaves

**DOI:** 10.1155/2021/8375961

**Published:** 2021-12-28

**Authors:** Chong Li, Yongpeng He, Yue Yang, Yuting Gou, Shuting Li, Rui Wang, Shi Zeng, Xin Zhao

**Affiliations:** ^1^Chongqing Collaborative Innovation Center for Functional Food, Chongqing Engineering Research Center of Functional Food, Chongqing Engineering Laboratory for Research and Development of Functional Food, Chongqing University of Education, Chongqing 400067, China; ^2^Department of Food and Nutrition, College of Medical and Life Science, Silla University, Busan 46958, Republic of Korea; ^3^Chongqing Key Laboratory of Translational Research for Cancer Metastasis and Individualized Treatment, Chongqing University Cancer Hospital & Chongqing Cancer Institute & Chongqing Cancer Hospital, Chongqing 400030, China; ^4^College of Biological and Chemical Engineering, Chongqing University of Education, Chongqing 400067, China; ^5^Department of Neurosurgery, People's Hospital of Chongqing Banan District, Chongqing 401320, China

## Abstract

This study is aimed at identifying the bioactive components in lotus leaf flavonoid extract (LLFE) and analyzing the antioxidant and anti-inflammatory activities of LLFE *in vitro* and *in vivo*. The flavonoids in LLFE were determined by UHPLC-MS/MS. The effect of LLFE on damaged 293T cells (H_2_O_2_, 0.3 mmol/L) was determined by MTT assay, and the activity of antioxidant enzymes was measured by kits. We studied the antioxidant and anti-inflammatory effects of LLFE on D-Gal/LPS (30 mg/kg·bw and 3 *μ*g/kg·bw)-induced aging mice. We also evaluated the main organ index, pathological changes in the liver, lung, and kidney, liver function index, biochemical index, cytokine level, and mRNA expression level in serum and liver. The results showed that LLFE contains baicalein, kaempferol, kaempferid, quercetin, isorhamnetin, hyperoside, lespenephryl, and rutin. LLFE reduced the oxidative damage sustained by 293T cells, increased the levels of SOD, CAT, GSH, and GSH-Px, and decreased the level of MDA. The animal studies revealed that LLFE reduced oxidative damage and inflammation in injured mice, inhibited increases in AST, ALT, MDA, and NO, increased SOD, CAT, GSH, and GSH-Px levels, upregulated anti-inflammatory cytokines IL-10 and IL-12, and downregulated proinflammatory cytokines IL-6, IL-1*β*, TNF-*α*, and IFN-*γ*. Furthermore, the expression of antioxidant- and anti-inflammatory-related mRNA was consistent with the above results.

## 1. Introduction

Homeostasis exists between prooxidation and antioxidation in the human body, but this balance is not stable and eventually induces oxidative stress due to various factors [1]. Oxidative stress occurs when there is an accumulation of high levels of reactive oxygen species (ROS). Biological components such as DNA, protein, and lipids in the human body are oxidized and modified by reactive oxygen free radicals or combined with other forms of free radicals or cross-linked, and these changes can induce cardiovascular disease, neurodegeneration, inflammatory disease, or cancer [2].

The establishment of a successful and reliable model of oxidative stress and inflammation is of great significance for the research and screening of antioxidant and anti-inflammatory drugs and functional foods. In cell models, hydrogen peroxide (H_2_O_2_) is an important ROS that is produced in almost all oxidative stress states. It easily penetrates cell membranes and reacts with intracellular Fe^2+^ through the Fenton reaction to effectively oxidize a variety of organic substances and form free radicals. Moreover, H_2_O_2_ is an important tool for building an *in vitro* oxidized cell model because it is easy to obtain and stable [3].

In animal models, excessive intake of D-galactose (D-Gal) will produce many free radicals and increases in peroxide and oxidation products, resulting in oxidative damage and inflammation [4]. Lipopolysaccharide (LPS) can mediate the innate immune response. High doses can cause fever, microcirculation disorders, endotoxin shock, and disseminated intravascular coagulation and other diseases; low doses can lead to the production of cytokines such as tumor necrosis factor and interleukin [5]. D-Gal can enhance the susceptibility of organisms to LPS, while LPS can increase the toxicity of D-Gal to organs and tissues. The acute and chronic organ injury induced by the combination of D-Gal and LPS has been applied in practical research, although the mechanism is still unclear [6].

In recent years, there has been significant progress in Chinese medicine in the research of antioxidant and anti-inflammatory effects. Many Chinese medicines (such as *Ginkgo biloba* [7], ginseng (*Panax ginseng*) [8], and reishi (*Ganoderma lucidum*) [9]) have been shown to have varying degrees of antioxidant components, which can prevent the chain reaction oxidation of unsaturated fatty acids by free radicals, enhance the activity of antioxidant enzymes, and inhibit expression of inflammatory cytokines in the organism.

Lotus (*Nelumbo nucifera* Gaertn.) is an aquatic plant that is cultivated in most parts of southern China and is widely used in food and drugs [10]. As a traditional Chinese medicine (TCM), dried lotus leaf has the functions of removing toxins, inhibiting bacteria, relieving constipation, and hemostasis, and it is an important component in a variety of lipid-lowering compound preparations. As a natural edible resource, lotus leaf has been developed into a variety of weight-loss and lipid-lowering teas or other antioxidant health drinks [11].

Modern pharmacological studies show that the flavonoids and alkaloids found in lotus leaf are two major types of chemical components with obvious biological activity and physiological functions. Flavonoids and alkaloids exert many physiological roles, including antiallergic, antibacterial, antioxidant, anti-inflammatory, and anticancer, and they are also used to decrease the amounts of circulating blood lipids and treat cardiovascular diseases [12–15]. In this study, lotus leaf was analyzed to determine its potential flavonoid bioactive components, and its antioxidant and anti-inflammatory effects were evaluated by cell and animal experiments, so as to provide a reference for the edible and medicinal value of lotus leaf.

## 2. Materials and Methods

### 2.1. Preparation of LLFE

Twenty grams of dried lotus leaves (produced in Anhui, China) was crushed, 100 mL of 70% ethanol was added according to the ratio of lotus leaf to ethanol (1 : 10 (*m* : v)), and the solution was incubated for 3 h in a 60°C water bath. After cooling, the above liquid was filtered to obtain the extraction solution. The filter residue was extracted again, and the crude extract of lotus leaf flavonoids was obtained by combining the two extraction solutions. To purify the crude extract, FL-3 macroporous resin containing the above crude extract was eluted with 70% ethanol, and the elution solution underwent rotary evaporation to remove the ethanol and water in the solution. Finally, the LLFE was obtained, which was dried at 60°C for 48 h, and then stored in a desiccator.

### 2.2. Ultrahigh Performance Liquid Chromatography-Tandem Mass Spectrometry (UHPLC-MS/MS) Analysis of LLFE

*Standard solution configuration*: 1 mg/mL standard stock solutions of baicalein, kaempferol, lespenephryl, kaempferid, isorhamnetin, rutin, hyperoside, and quercetin (Beijing Putian Tongchuang Biotechnology Co., Ltd. Beijing, China) were prepared with 50% methanol and stored at 2-8°C. The 8 standard stock solutions were gradually diluted with 50% methanol to 1 *μ*g/mL until use.

*Sample solution configuration*: 10 mg of LLFE and 1 mL of dimethyl sulfoxide (DMSO) were ultrasonicated for 10 minutes in 60°C water bath, diluted by 50% methanol to 2 mg/mL, and sterilized by filtering through a membrane (0.22 *μ*m) for later use.

*UHPLC parameters*: PerkinElmer QSight™ LX50 UHPLC system; PerkinElmer Brownlee SPP C18 column (2.1 × 100 mm, 2.7 *μ*m); sample size: 5 *μ*L; and column oven temperature: 40°C. The specific conditions are shown in Table 1.

*MS parameters*: PerkinElmer QSight™ 210 triple quadrupole mass spectrometry system; electrospray ion (ESI) source; multiple-reaction monitoring (MRM); nebulizer gas: 180; HSID temperature: 250°C; source temperature: 300°C; positive ion mode; drying gas (N_2_): 100; and electrospray V pos/neg: 5500 V.

The mass spectrometry parameters for the 8 types of standard samples were optimized using an injection pump to directly inject standard solution into the ion source. *Ion source conditions*: 20 *μ*m/min, 1 *μ*g/mL. In positive ionization mode, the parent ion peak was obtained by first-order mass spectrometry (Q1 scan), and the fragment ions were obtained by secondary scanning (product scan) of the target ion. The two fragment ions with the most significant responses were selected to obtain optimized conditions (Table 2) and analyzed.

### 2.3. LLFE Treatment of 293T Human Embryonic Kidney Cells Induced by H_2_O_2_

The *in vitro* antioxidative damage effect of LLFE was evaluated by the MTT (3-(4,5-dimethylazol-2-yl)-2,5-diphenyltetrazolium bromide) method. This assay was performed by seeding 160 *μ*L of 293T cell suspension (1 × 10^4^ cells/mL) in a 96-well plate and culturing (37°C, 5% CO_2_, 24 h) until cell adherence. Then, H_2_O_2_ and different concentrations of LLFE were added (Table 3). To each well, MTT (5 mg/mL, 20 *μ*L) was added, and the plate was incubated at 37°C for another 4 h. The upper nutrient base was discarded, DMSO (37°C, 150 *μ*L) was added, the cells were incubated for half an hour, and then, the optical density (OD) value was measured at 490 nm [16].

### 2.4. Quantitation of Malondialdehyde (MDA), Superoxide Dismutase (SOD), Catalase (CAT), Glutathione (GSH), and Glutathione Peroxidase (GSH-Px) in LLFE-Treated 293T Cells That Underwent Oxidative Damage Induced by H_2_O_2_ Treatment

Oxidation model (H_2_O_2_, 0.3 mmol/L) was prepared in a 6-well plate (1 × 10^5^ cells/mL) and then treated with LLFE (100 or 200 *μ*g/mL). After the cells were treated according to the above methods, the medium was collected and the LDH level in the medium was determined per the kit's instructions. After removing the medium and washing with precooled PBS, the cells were detached by trypsin (200 *μ*L) and centrifuged (4000 rpm, 15 minutes) to remove them and then lysed by an ultrasonic cell breaker in an ice water bath (vibrated for 3-5 s, repeated three times at a 4 s interval). The amounts of oxidation-related indicators in the cells were determined per the instructions (Nanjing Jiancheng Bioengineering Institute, Jiangsu, China).

### 2.5. Grouping and Establishment of Oxidative Stress and an Inflammation Model Using Kunming Mice

**Figure 1:**
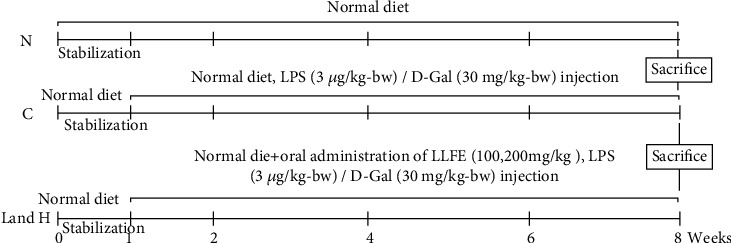
Mice experimental design. N: normal mice; C: control mice; L: low-dose LLFE-treated mice; H: high-dose LLFE-treated mice.

All mice (Kunming, male, 6-week-old) were fed a standard diet (Table 4) [17] and provided with drinking water ad libitum. After adapting to the new environment for 1 week, 4 groups of mice were formed, with 10 mice in each group. During the 8-week experimental period, the normal group mice were left untreated. The mice from the control and treatment groups were injected intraperitoneally with D-Gal/LPS daily. In addition, the treatment groups received different doses of LLFE (100 or 200 mg/kg) daily that were administered orally. The 0.1 mL/10 g·bw was administered by injection or oral dose (Figure 1). The protocol for these experiments was approved by the Ethics Committee of Chongqing Collaborative Innovation Center for Functional Food (202101006B, Chongqing, China) and followed the national standard of the People's Republic of China (GB/T 35892-2018) laboratory animal guidelines for ethical review of animal welfare.

### 2.6. Collection of Experimental Samples from Mice

After fasting for 18 hours, blood was collected from the retroorbital sinus (4°C; after retaining for 0.5 h, the blood was centrifuged for 15 min). The liver, lung, and kidney were weighed and then dissected. Portions were used for slice preparation and serum, and the remaining parts were stored at -80°C for subsequent experiments.

### 2.7. Organizational Observations, Serum Biochemical Indicators, and Liver Gene Expression in Mice

The liver, lung, and kidney tissues were stained by hematoxylin and eosin (H&E) to observe the pathological changes through an optical microscope [18].

The kit method was used to detect serum MDA, CAT, GSH, GSH-Px, aspartate transaminase (AST), alanine transaminase (ALT), nitric oxide (NO), SOD (Nanjing Jiancheng, Jiangsu, China), and tumor necrosis factor- (TNF-) *α*, interferon- (IFN-) *γ*, interleukin- (IL-) 1*β*, IL-6, IL-12, and IL-10 (Abcam, Cambridge, MA, USA) levels.

After homogenization of liver tissues, total RNA was extracted (TRIzol™ reagent) and reverse transcribed into cDNA (Thermo Fisher, Waltham, MA, USA). The 2^-∆∆CT^ method was adopted to assess gene levels (Table 5) [18].

### 2.8. Software and Data Analysis Method

The data were statistically analyzed by analysis of variance (ANOVA) and Duncan's multiple range tests after the Shapiro-Wilk test for normal distribution (*P* < 0.05) through the SPSS statistical software (version 20.0, Chicago, USA).

## 3. Results

### 3.1. LLFE Chemical Composition

**Figure 2:**
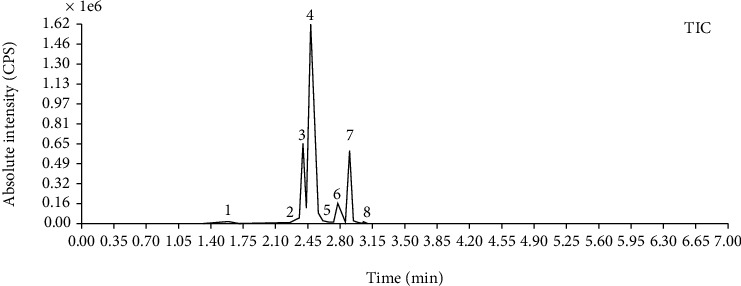
LLFE ion chromatography. 1: lespenephryl, 2: kaempferid, 3: rutin, 4: quercetin, 5: hyperoside, 6: kaempferol, 7: isorhamnetin, and 8: baicalein.

Compared with the retention time and mass spectrometry of the chemical standard, the target compounds were qualitatively and quantitatively analyzed. Eight flavonoids were identified from LLFE (Figure 2 and Table 6).

### 3.2. Effects of LLFE on the Survival Ability of 293T Cells and Damaged 293T Cells

**Figure 3:**
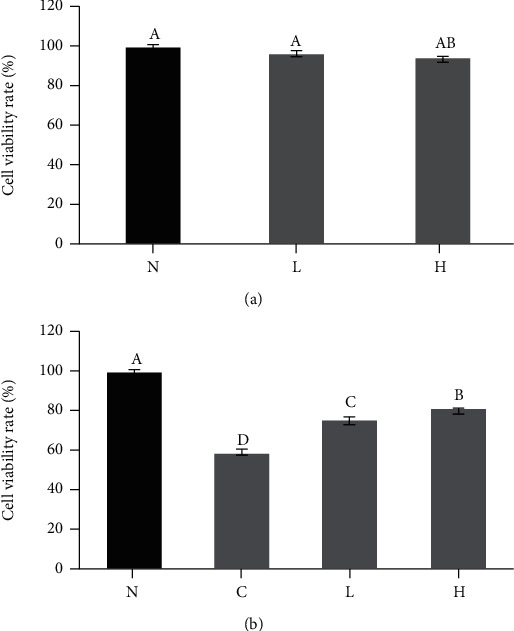
LLFE has no (a) significant toxic effect on 293T cells and (b) increase viability of damaged 293T cells. N: 0.9% NaCl-treated 293T cells; C: H_2_O_2_-treated 293T cells; L: H_2_O_2_ and low-dose LLFE-treated 293T cells; H: H_2_O_2_ and high-dose LLFE-treated 293T cells. ^a-d^Different letters in the same image have significance (*P* < 0.05) based on Duncan's multirange test.

The viability of 293T cells treated with LLFE (100 or 200 *μ*g/mL) exceeded 95% (Figure 3(a)), indicating that there was no significant lethal effect of LLFE (0-200 *μ*g/mL) on 293T cells. The H_2_O_2_-treated-cell viability was significantly lower compared to normal cells. The cell viability was significantly increased after LLFE (100 or 200 *μ*g/mL) treatment, and a more significant effect was observed for the high concentration of LLFE (Figures 3(b), *P* < 0.05).

### 3.3. Effect of LLFE on Antioxidant Indexes of Damaged 293T Cells

**Figure 4:**
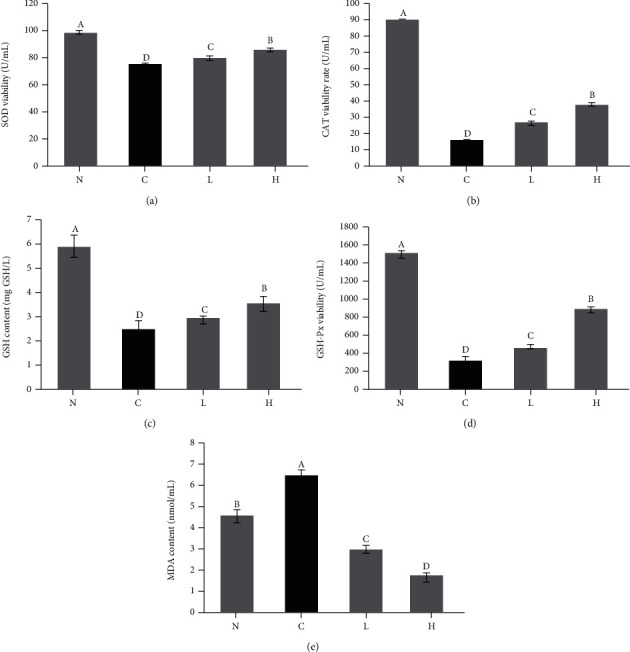
Effects of LLFE on antioxidant index levels in 293T cells induced by H_2_O_2_. N: 0.9% NaCl-treated 293T cells; C: H_2_O_2_-treated 293T cells; L: H_2_O_2_ and low-dose LLFE-treated 293T cells; H: H_2_O_2_ and high-dose LLFE-treated 293T cells. ^a-d^Different letters in the same image have significance (*P* < 0.05) based on Duncan's multirange test.

The levels of SOD, CAT, GSH, and GSH-Px in the control group were decreased, while MDA level was increased (Figure 4, *P* < 0.05) compared to the normal group. LLFE inhibited the deterioration of this situation and alleviated the oxidative damage of H_2_O_2_-induced 293T cells.

### 3.4. Organ Index in Mice

Organ indexes (liver, lung, and kidney) were reduced (Table 7, *P* < 0.05) after long-term D-Gal/LPS stimulation, and organ atrophy was suppressed to a certain extent after LLFE treatment.

### 3.5. Histological Analyses

**Figure 5:**
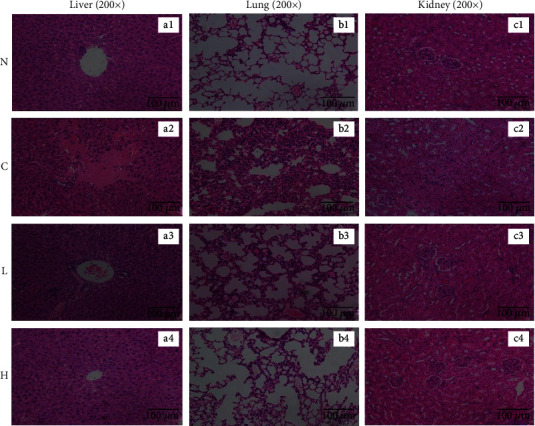
(a1–a4) Effects of LLFE on the histomorphology of the liver, (b1–b4) lung, and (c1–c4) kidney in mice. N: normal mice; C: control mice; L: low-dose LLFE-treated mice; H: high-dose LLFE-treated mice.

Normal hepatocytes exhibited regular morphology, clear structure, and orderly arrangement of hepatocyte cords, with radial distribution (Figure 5(a1)). The control hepatocytes showed the opposite state—the arrangement of hepatocytes was disordered, the central vein was abnormal, the boundary was unclear, the cells were swollen, the staining was uneven, and infiltration of inflammatory cells was observed (Figure 5(a2)). After LLFE treatment, the hepatocyte morphology and arrangement appeared more normal, and the inflammation was reduced (Figures 5(a3) and 5(a4)).

Figures 5(b1)–5(b4) prove that the normal alveolar walls were intact, the alveolar cavity was clear, there was no edema in the alveolar and pulmonary interstitium, and there were no inflamed cells. There were pathological changes in control lung tissue, such as destruction of alveolar structure, edema of the alveolar wall, thickening of the pulmonary interstitium, atrophy of the alveolar cavity, and infiltration of a large number of inflammatory cells. After LLFE treatment, the alveolar structure was more complete, and the infiltration of inflammatory cells was significantly reduced.

As shown in Figures 5(c1)–5(c4), the shapes of the normal glomeruli and renal tubules were regular, and the epithelial cells of renal tubules were arranged in an orderly fashion, with a clear structure and uniform cytoplasm. In the control group, the renal tubular epithelial cells swelled and fell off, with vacuolar degeneration, glomerular shrinkage, endothelial cell degeneration, and inflammatory cell infiltration. After LLFE treatment, the renal tissue structure returned to normal, and the inflammatory cell infiltration was alleviated by different degrees.

### 3.6. Liver Function Indexes in Mouse Serum

**Figure 6:**
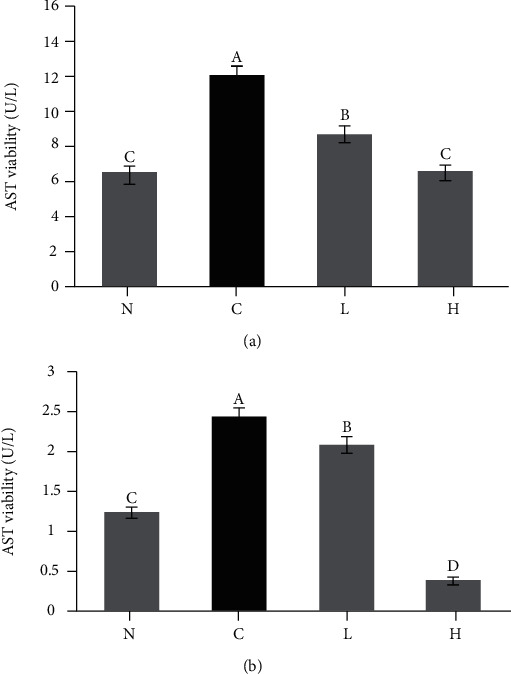
The (a) AST and (b) ALT activity in mouse serum. N: normal mice; C: control mice; L: low-dose LLFE-treated mice; H: high-dose LLFE-treated mice. ^a-d^Different letters in the same image have significance (*P* < 0.05) based on Duncan's multirange test.

The aspartate AST and alanine ALT activity in the control serum was higher compared to the other three groups (Figure 6, *P* < 0.05). After LLFE treatment, the serum AST and ALT values decreased by different degrees.

### 3.7. Biochemical Indexes in Mouse Serum

**Figure 7:**
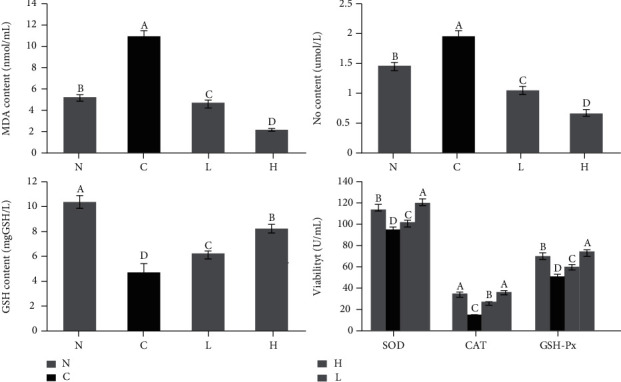
Effects of LLFE on the oxidation-related indicators of mouse serum. N: normal mice; C: control mice; L: low-dose LLFE-treated mice; H: high-dose LLFE-treated mice. ^a-d^Different letters in the same image have significance (*P* < 0.05) based on Duncan's multirange test.

**Figure 8:**
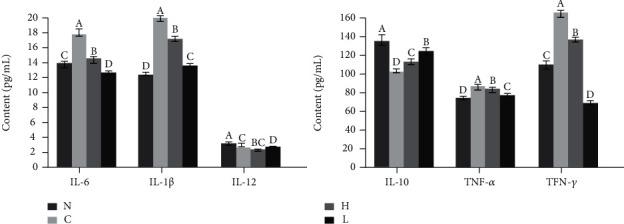
Effects of LLFE on the inflammation-related cytokines of mouse serum. N: normal mice; C: control mice; L: low-dose LLFE-treated mice; H: high-dose LLFE-treated mice. ^a-d^Different letters in the same project have significance (*P* < 0.05) based on Duncan's multirange test.

The enzymatic activities of SOD, CAT, GSH, and GSH-Px in the control serum decreased, while the levels of MDA and NO increased after long-term D-Gal/LPS stimulation (Figure 7, *P* < 0.05). The control serum IL-6, IL-1*β*, TNF-*α*, and IFN-*γ* expression increased, while the expression of IL-10 and IL-12 decreased (Figure 8, *P* < 0.05). LLFE treatment effectively enhanced the antioxidant and anti-inflammatory enzymes and compounds in mouse serum, and there was greater effectiveness with the high dose of LLFE.

### 3.8. Gene Expression in Mouse Liver Tissue

**Figure 9:**
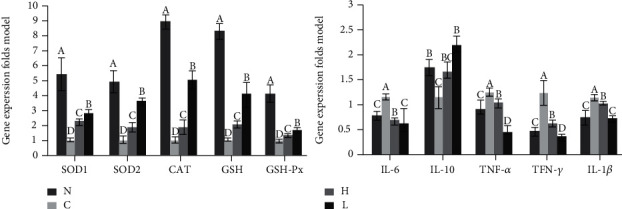
Effects of LLFE on mRNA expression in mouse liver tissue. N: normal mice; C: control mice; L: low-dose LLFE-treated mice; H: high-dose LLFE-treated mice. ^a-d^Different letters in the same project have significance (*P* < 0.05) based on Duncan's multirange test.

The expression of SOD1, SOD2, CAT, GSH, GSH-Px, and IL-10 in control liver tissues decreased, while the expression of IL-1*β*, IL-6, TNF-*α*, and IFN-*γ* increased (Figure 9, *P* < 0.05) after D-Gal/LPS stimulation. After LLFE treatment, oxidative stress and inflammatory gene expression were significantly decreased, while the expression of antioxidant and anti-inflammatory genes was enhanced.

**Figure 10:**
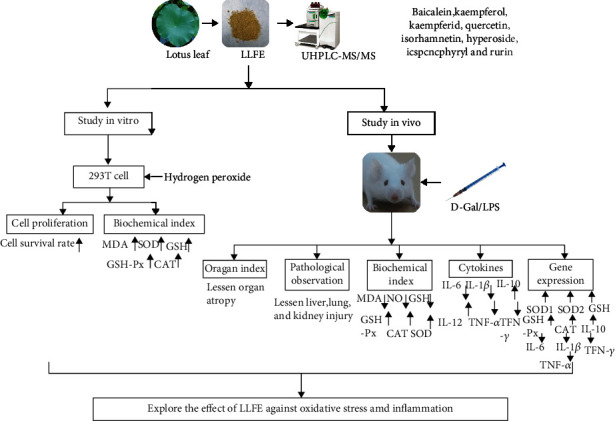
A summary of the framework and mechanism in this research. LLFE: lotus leaf flavonoid extract.

Based on the above analysis of our results, we conclude that LLFE exhibits a strong protective effect on 293T cells and mice from oxidative damage and inflammation (Figure 10).

## 4. Discussion

Lotus leaf is a natural food and TCM that has many functions, including inhibition of oxidation and inflammation, lowering of lipids, and resisting viruses [19, 20]. The chemical composition of lotus leaf is relatively complex. In addition to many carbohydrates, lipids, proteins, tannins, and other common chemical components, it also contains polysaccharides, organic acids, alkaloids, and flavonoids with clear biological and physiological functions. In particular, lotus leaf contains abundant flavonoids, mostly in the form of glycosides, and most of the aglycones are quercetin and kaempferol [21, 22]. Therefore, in this study, we used 70% ethanol to extract the total flavonoids in lotus leaves and identified 8 active substances in LLFE through UHPLC-MS/MS.

There is a notable amount of quercetin in lotus leaves, and this compound possesses effective antioxidant, anti-inflammatory, analgesic, hypoglycemic, antiviral, and antitumor action [23]. Kaempferol is a flavonoid and is widely found in many vegetables and fruits, including lotus leaves. Studies have shown that kaempferol exhibits neuroprotection and protein kinase inhibition action, and it also inhibits platelet aggregation [24]. Rutin is a natural flavonoid glycoside that assists the action of oxidoreductase, acting on the thyroid and adrenal glands, acting in synergy with vitamin C in the body to protect blood vessels, and also promoting cell proliferation and antithrombotic action, antilipid peroxidation, and scavenging of free radicals [25]. Baicalein is a xanthine oxidase inhibitor that can inhibit allergic reactions. After entering the body, it is quickly converted into baicalein and other metabolites in the blood, which can increase cerebral blood circulation [26].

Others, such as isorhamnetin, expand the cardiovascular system, inhibit thrombosis, and inhibit adipogenesis by interfering with the differentiation of adipose stem cells [27]. Hyperoside exerts a local analgesic effect, inhibits myocardial and cerebral infarction, and reduces cholesterol and protein assimilation [28]. LLFE embodies various physiological functions due to these active compounds.

First, we analyzed the ability of LLFE to respond to H_2_O_2_-damaged 293T cells *in vitro*. H_2_O_2_ is an important tool for oxidative stress research, but it has a bidirectional effect on cells due to the final concentration of H_2_O_2_ and the treatment time used determine the success of the 293T cell oxidative stress model [3]. We finally determined that the oxidation model after the resulting damage caused by H_2_O_2_ (0.3 mmol/L, 4 h) was ideal because the level of MDA in 293T cells increased, while the level of antioxidant enzymes decreased. After LLFE treatment, the oxidative damage was suppressed, and the antioxidant capacity was enhanced in 293T cells.

Furthermore, we studied the health improvement that resulted from administration of LLFE to D-Gal/LPS-damaged mice from the liver pathological aspect and liver function indexes. D-Gal is a liver cell uridine phosphate interfering agent that depletes uridine phosphate by competing for the production of uridine galactose diphosphate, which subsequently limits the regeneration of organelles and the production and supplement of enzymes. As a result, organelles sustain damage, and the structure and function of liver cells become abnormal, which leads to metabolic disorders and causes liver cell degeneration and necrosis [4].

The study of Song et al. proved that D-Gal induction can lead to oxidative stress in mice [29]. However, the meta-analysis of Sadigh-Eteghad et al. showed that the clinical use of D-Gal is defective, and the induced oxidation state cannot completely mimic natural aging [30]. It should be noted that a low-dose inducer is futile because recovery is facile from the oxidative stress caused by it once the induction is stopped [31]. LPS is an endotoxin that can activate monocyte macrophages. It binds to the transmembrane receptor TLR4 on monocyte macrophages and endothelial cells mediated by binding protein and CD14. Then, the signal is transferred to the cell with the assistance of lymphocyte antigen 96. Through a complex intracellular signaling pathway, secretion of inflammatory factors occurs, thus causing liver cell injury [5]. LPS can also synergistically act with D-Gal to cause liver cell damage through endotoxemia and induce neutrophils to participate in degranulation of damaged liver cells. The synergistic reaction also releases oxygen free radicals that cause lipid peroxidation. D-Gal acts on liver parenchymal cells and vascular endothelial cells, causing severe cell damage or death [6].

Studies have shown that the liver injury and oxidative inflammation model of mice induced by the combination of D-Gal and LPS is more effective and more stable [32, 33]. Previous articles have shown that flavonoids such as puerarin [34] and betulin [35] effectively reverse D-Gal/LPS-induced oxidative inflammation, improve liver pathology, reduce serum ALT and AST levels, and reduce proinflammatory cytokine production in mice. Therefore, our study used a combination of these two drugs to induce a mouse model. We confirmed that the two drugs induced varying degrees of organ atrophy and weight loss. Histological observation also revealed structural damage and inflammatory cell infiltration, and similarly, elevated serum ALT and AST levels indicated an increase in liver deterioration, and LLFE treatment reversed these alterations [36].

In more depth, we explored the mechanism of LLFE on D-Gal/LPS-induced mice, examining oxidative stress and inflammation. MDA, the end product of lipid oxidation, can interfere with mitochondrial respiratory chain complexes and key enzymatic activities in mitochondria and also aggravate membrane damage. The amount of serum MDA indirectly reflects the degree of cell damage [37]. NO activates soluble guanylate cyclase to relax blood vessels. However, NO derived from inducible nitric oxide synthase (iNOS) and neuronal NOS confers neurotoxic effects [38]. Furthermore, studies have shown that there is a synergistic effect between CAT and SOD. SOD can decompose O_2_^−^ into H_2_O_2_, which can be further reduced by CAT to water [39]. GSH is a low-molecular-weight, nonenzymatic scavenger of lipid free radicals that works in tandem with GSH-Px. Selenium in GSH-Px can catalyze GSH to GSSG, reduce toxic peroxides to nontoxic hydroxyl compounds, and promote H_2_O_2_ decomposition [40].

Previous studies have shown that lotus leaf extract enhances the antioxidant capacity in alcohol-induced gastric injury mice [41] and high-fat diet-induced obese mice [42], including the ability to increase SOD, CAT, GSH, and GSH-PX and decrease the levels of MDA and NO. We further found that LLFE also significantly inhibits the levels of MDA and NO in D-Gal/LPS-induced mice and increases the levels of serum antioxidant enzymes and compounds.

The immune response of animals is divided into responses involving a variety of cytokines and includes Th1-mediated delayed hypersensitivity involved in cellular immunity and Th2-mediated immediate hypersensitivity in humoral immunity, both of which are derived from the common precursor cell Th0 [43]. IL-12 is an essential factor for Th1 cell differentiation, IFN-*γ* and IL-2 promote Th1 differentiation, and IFN-*γ* promotes the production of IL-12. Therefore, IL-12 and IFN-*γ* exert synergistic effects on the regulation of Th subset differentiation [44].

TNF-*α* is a systemic inflammatory cytokine mainly secreted by macrophages. TNF-*α* performs many functions, including preventing bacterial infections, regulating cell growth and the immune system, and participating in septic shock [45]. The IL-1 family is mainly related to innate immunity, which mainly manifests as the host's inflammatory response. IL-1*β* is secreted through an unconventional protein secretion pathway and participates in the regulation of IL-6 and TNF-*α*. Studies have shown that LPS and ATP can induce monocytes and macrophages to release IL-1*β* [46].

IL-10 inhibits inflammation and cellular immune responses, strengthens tolerance related to adaptive immunity and clearance, and inhibits proinflammatory factors produced by monocytes and macrophages. IL-6 is a pleiotropic cytokine that significantly changes in a variety of diseases, and it can be produced by macrophages, T lymphocytes, and B lymphocytes after being induced by viruses, endotoxin, and TNF. IL-6 can induce the differentiation of B cells to produce immunoglobulins, promote the proliferation and growth of T cells, and enhance the differentiation of blood cells and antitumor effects [47].

Published literature shows that lotus leaf reduces the levels of inflammatory cytokines IL-1*β*, TNF-*α*, IFN-*γ*, and IL-6, increases the levels of anti-inflammatory cytokines IL-4 and IL-10, and inhibits inflammation caused by high-fat diets accompanied by obesity [48]. Lotus leaf also inhibits the JNK/NF-*κ*B signaling pathway through the active compounds quercetin and catechin, thereby reducing the inflammatory response of macrophages [48]. In addition, lotus seedpod extract inhibits LPS-induced liver inflammation by downregulating the Toll-like receptor 4- (TLR4-) mediated NF-*κ*B and p38 signaling pathways [49]. In this study, LLFE treatment increased the anti-inflammatory level and decreased the proinflammatory level mentioned above in injured mice.

## 5. Conclusions

LLFE inhibited oxidative damage to 293T cells induced by H_2_O_2_, as well as the oxidative stress and inflammation damage induced by D-Gal/LPS in mice. It increased the levels of antioxidant enzymes and compounds in damaged 293T cells and damaged mice, reduced peroxide levels, and regulated the levels of related immune factors. LLFE exhibits strong antioxidant and anti-inflammatory abilities both *in vivo* and *in vitro*.

## Figures and Tables

**Table 1:** UHPLC conditions.

Time (min)	Flow rate (mL/min)	A phase (%)	B phase (%)	Shape
0	0.3	90	10	---
1	0.3	90	10	Linear
3	0.3	10	90	Linear
5	0.3	10	90	Linear
5.5	0.3	90	10	Linear
7	0.3	90	10	Linear

A phase: 0.01% (v/v) formic acid water and B phase: acetonitrile.

**Table 2:** Retention time and mass spectrometry parameters for 8 flavonoids.

Component	Parent ion (m/z)	Daughter ion (m/z)	Collision energy/V	Entrance voltage/V
Baicalein	271.1	123.1^∗^	-51	11
		253.1	-90	12
Kaempferol	287.1	153.1^∗^	-51	63
		213.1	-52	47
Kaempferid	301.1	286.1	-98	16
		165.1^∗^	-55	20
Quercetin	303.2	229.1^∗^	-51	55
		257.1	-97	59
Isorhamnetin	317.3	88.1^∗^	-51	24
		256.4	-98	2
Hyperoside	465.1	106.2^∗^	-79	25
		153	-10	21
Lespenephryl	579.3	287.1^∗^	-52	8
		433.1	-95	13
Rutin	611.2	303.1^∗^	-82	14
		465.2	-99	19

^∗^Quantitative ion.

**Table 3:** Effect of LLFE on the survival rate of 293T cells and H_2_O_2_-injured 293T cells.

Group	N	C	L	H
A	0.9% NaCl	---	100 *μ*g/mL LLFE (24 h)	200 *μ*g/mL LLFE (24 h)
B	0.9% NaCl	0.3 mmol/L H_2_O_2_ (4 h)	0.3 mmol/L H_2_O_2_ (4 h)+100 *μ*g/mL LLFE (24 h)	0.3 mmol/L H_2_O_2_ (4 h)+100 *μ*g/mL LLFE (24 h)

A: toxicity of LLFE on 293T cells, B: effect of LLFE on H_2_O_2_-induced 293T cells. N: 0.9% NaCl-treated 293T cells; C: H_2_O_2_-treated 293T cells; L: H_2_O_2_ and low-dose LLFE-treated 293T cells; H: H_2_O_2_ and high-dose LLFE-treated 293T cells. The dose of 0.9% NaCl, LLFE, and H_2_O_2_ is all 20 *μ*L.

**Table 4:** Composition of normal diet.

Product	Ingredient	Mass/%
Normal diet	Wheat flour	25
	Oatmeal	25
	Corn flour	25
	Soybean flour	10
	Fish meal	8
	Hog bone powder	4
	Yeast powder	2
	Refined salt	1

**Table 5:** Primer and RT-qPCR conditions.

Primer name	Sequence
*SOD1*	F: 5′-AACCAGTTGTGTTGTGAGGAC-3′
	R: 5′-CCACCATGTTTCTTAGAGTGAGG-3′
*SOD2*	F: 5′-CAGACCTGCCTTACGACTATGG-3′
	R: 5′-CTCGGTGGCGTTGAGATTGTT-3′
*CAT*	F: 5′-GGAGGCGGGAACCCAATAG-3′
	R: 5′-GTGTGCCATCTCGTCAGTGAA-3′
*GSH*	F: 5′-TATCAGAGGCGGGAAATCTCTT-3′
	R: 5′-ATTCTTGCTTCGGCCACATAC-3′
*GSH-Px*	F: 5′-GTCGGTGTATGCCTTCTCGG-3′
	R: 5′-AGAGAGACGCGACATTCTCAAT-3′
*IL-10*	F: 5′-CTTACTGACTGGCATGAGGATCA-3′
	R: 5′-GCAGCTCTAGGAGCATGTGG-3′
*IL-6*	F: 5′-CTGCAAGAGACTTCCATCCAG-3′
	R: 5′-AGTGGTATAGACAGGTCTGTTGG-3′
*TNF-α*	F: 5′-CAGGCGGTGCCTATGTCTC-3′
	R: 5′-CGATCACCCCGAAGTTCAGTAG-3′
*IFN-γ*	F: 5′-GGCCTAGCTCTGAGACAATGAAC-3′
	R: 5′-TGACCTCAAACTTGGCAATACTC-3′
*IL-1β*	F: 5′-GAAATGCCACCTTTTGACAGTG-3′
	R: 5′-TGGATGCTCTCATCAGGACAG-3′
*β-Actin*	F: 5′-GAGAAAATCTGGCACCACACCT-3′
	R: 5′-GCACAGCCTGGATAGCAACGTA-3′

Operating temperature: predenaturation 95°C 30 s, PCR reaction 95°C 5 s and 60°C 30 s, and 40 cycles.

**Table 6:** Results of eight flavonoids in LLFE.

Name	Molecular mass	Molecular formula	Structural formula	Retention time (min)	Content (ng/mL)
Lespenephryl	578.51	C_27_H_30_O_14_	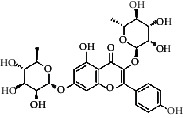	1.55	683.037
Kaempferid	300.26	C_16_H_12_O_6_	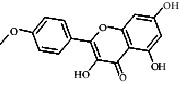	2.31	562.891
Rutin	610.51	C_27_H_30_O_16_	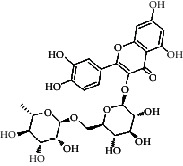	2.39	490.072
Quercetin	302.24	C_15_H_10_O_7_	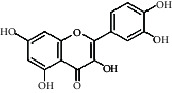	2.48	7046.494
Hyperoside	464.38	C_21_H_20_O_12_	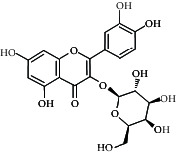	2.52	88.845
Kaempferol	286.24	C_15_H_10_O_6_	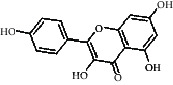	2.72	2436.970
Isorhamnetin	316.26	C_16_H_12_O_7_	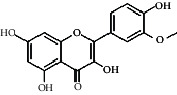	2.90	567.088
Baicalein	270.24	C_15_H_10_O_5_	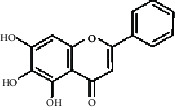	3.02	306.363

**Table 7:** Effects of LLFE on mouse organ indexes.

Groups	Lung	Liver	Kidney
N	5.19 ± 0.30^b^	40.81 ± 3.81^b^	13.29 ± 1.21^b^
C	4.9 ± 0.39^c^	37.47 ± 3.65^c^	12.66 ± 1.56^c^
L	5.27 ± 1.26^b^	40.38 ± 2.75^b^	13.21 ± 0.81^b^
H	6.45 ± 3.8^a^	43.73 ± 6.89^a^	14.17 ± 1.21^a^

N: normal mice; C: control mice; L: low-dose LLFE-treated mice; H: high-dose LLFE-treated mice. ^a-d^Different letters in the same column have significance (*P* < 0.05) based on Duncan's multirange test. ^A^Mean ± standard deviation (SD). Organ coefficient (mg/g) = organ weight (mg)/body weight (g).

## Data Availability

The datasets generated for this study are available upon request to the corresponding author.

## References

[1] Sohal R. S., Allen R. G. (1990). Oxidative stress as a causal factor in differentiation and aging: a unifying hypothesis. *Experimental Gerontology*.

[2] Radak Z., Zhao Z., Koltai E., Ohno H., Atalay M. (2013). Oxygen consumption and usage during physical exercise: the balance between oxidative stress and ROS-dependent adaptive signaling. *Antioxidants & Redox Signaling*.

[3] Bian Y. Y., Guo J., Majeed H. (2015). Ferulic acid renders protection to HEK293 cells against oxidative damage and apoptosis induced by hydrogen peroxide. *In Vitro Cellular & Developmental Biology-Animal*.

[4] Zhang Z. F., Fan S. H., Zheng Y. L. (2009). Purple sweet potato color attenuates oxidative stress and inflammatory response induced by d-galactose in mouse liver. *Food and Chemical Toxicology*.

[5] Paik Y. H., Schwabe R. F., Bataller R., Russo M. P., Jobin C., Brenner D. A. (2003). Toll-like receptor 4 mediates inflammatory signaling by bacterial lipopolysaccharide in human hepatic stellate cells. *Hepatology*.

[6] Liu H., Zhang W., Dong S. (2015). Protective effects of sea buckthorn polysaccharide extracts against LPS/d-GaLN-induced acute liver failure in mice via suppressing TLR4-NF-*κ*B signaling. *Journal of Ethnopharmacology*.

[7] Ren Q., Chen J., Ding Y. (2019). In vitro antioxidant and immunostimulating activities of polysaccharides from _Ginkgo biloba_ leaves. *International Journal of Biological Macromolecules*.

[8] Chen F., Huang G. L. (2019). Antioxidant activity of polysaccharides from different sources of ginseng. *International Journal of Biological Macromolecules*.

[9] Xu Y., Zhang X., Yan X. H. (2019). Characterization, hypolipidemic and antioxidant activities of degraded polysaccharides from *Ganoderma lucidum*. *International Journal of Biological Macromolecules*.

[10] Yang J. Y., Hu L., Yang X. (2007). Research progress of flavonoids in lotus leaves. *Food ence (in Chinese)*.

[11] He L. P. (2017). Development of lotus leaf tea beverage for lowing blood lipids. *Cereals and oils (in Chinese)*.

[12] Temviriyanukul P., Sritalahareuthai V., Promyos N. (2020). The effect of sacred lotus (*Nelumbo nucifera*) and its mixtures on phenolic profiles, antioxidant activities, and inhibitions of the key enzymes relevant to Alzheimer's disease. *Molecules*.

[13] Lee K., Kim J., Lee N., Park S., Cho H., Chun Y. (2015). Effects of potato and lotus leaf extract intake on body composition and blood lipid concentration. *Journal of Exercise Nutrition & Biochemistry*.

[14] Su D., Li N., Chen M. (2018). Effects ofin vitrodigestion on the composition of flavonoids and antioxidant activities of the lotus leaf at different growth stages. *Journal of food technology*.

[15] Yang D.-H., Lou Z.-H., Cheng B., Zhang G.-J., Wang Y.-P., Xu H. (2016). Effects of lotus leaf on inflammatory factors and liver AdipoR2 expressions in rats with NAFLD induced by high fat diet and high glucose. *China Journal of Chinese Materia Medica (in Chinese)*.

[16] Li C., Huang G., Tan F., Zhou X., Mu J., Zhao X. (2019). In vitro analysis of antioxidant, anticancer, and bioactive components of apocynum venetum tea extracts. *Journal of Food Quality*.

[17] Li C., Liu H., Yang J., Mu J., Wang R., Zhao X. (2020). Effect of soybean milk fermented withLactobacillus plantarumHFY01 isolated from yak yogurt on weight loss and lipid reduction in mice with obesity induced by a high-fat diet. *RSC Advances*.

[18] Yi R. K., Tan F., Liao W. (2019). Isolation and identification of *Lactobacillus plantarum* HFY05 from natural fermented yak yogurt and its effect on alcoholic liver injury in mice. *Microorganisms*.

[19] Huang K. Y., Zhang Z. G. (2008). The information on the research of pharmaceutical used part of Heye (lotus leaf). *China Pharmaceuticals (in Chinese)*.

[20] Su P. Q., Su G. L. (1996). Medicinal and clinical significance of lotus. *Journal of Chengde medical college (in Chinese)*.

[21] Chen X., Qi J. (2015). Flavonoids and alkaloids in lotus leaves. *Chinese Journal of Experimental Traditional Medical Formulae (in Chinese)*.

[22] Ye L. H., He X. X., Yan M. Z., Chang Q. (2014). Identification of in vivo components in rats after oral administration of lotus leaf flavonoids using ultra fast liquid chromatography with tandem mass spectrometry. *Analytical Methods*.

[23] Kawabata K., Mukai R., Ishisaka A. (2015). Quercetin and related polyphenols: new insights and implications for their bioactivity and bioavailability. *Food & Function*.

[24] Lin C., Wu F., Zheng T., Wang X., Chen Y., Wu X. (2019). Kaempferol attenuates retinal ganglion cell death by suppressing NLRP1/NLRP3 inflammasomes and caspase-8 via JNK and NF-*κ*B pathways in acute glaucoma. *Eye*.

[25] Sun J., Wang H., Liu B. (2017). Rutin attenuates H_2_O_2_-induced oxidation damage and apoptosis in Leydig cells by activating PI3K/Akt signal pathways. *Biomedicine & Pharmacotherapy*.

[26] Yin H., Huang L., Ouyang T., Chen L. (2018). Baicalein improves liver inflammation in diabetic db/db mice by regulating HMGB1/TLR4/NF-*κ*B signaling pathway. *International Immunopharmacology*.

[27] Wang J., Gong H. M., Zou H. H., Liang L., Wu X.‑. Y. (2018). Isorhamnetin prevents H_2_O_2_-induced oxidative stress in human retinal pigment epithelial cells. *Molecular Medicine Reports*.

[28] Chen Y., Ye L., Li W., Li D., Li F. (2018). Hyperoside protects human kidney‑2 cells against oxidative damage induced by oxalic acid. *Molecular Medicine Reports*.

[29] Song X., Bao M. M., Li D. D., Li Y. M. (1999). Advanced glycation in D-galactose induced mouse aging model. *Mechanisms of Ageing and Development*.

[30] Sadigh-Eteghad S., Majdi A., McCann S. K., Mahmoudi J., Vafaee M. S., Macleod M. R. (2017). D-galactose-induced brain ageing model: a systematic review and meta-analysis on cognitive outcomes and oxidative stress indices. *PLoS One*.

[31] Hadzi-Petrushev N., Stojkovski V., Mitrov D., Mladenov M. (2015). D-galactose induced changes in enzymatic antioxidant status in rats of different ages. *Physiological Research*.

[32] Li F. F., Miao L. Y., Sun H., Zhang Y., Bao X., Zhang D. (2017). Establishment of a new acute-on-chronic liver failure model. *Acta Pharmaceutica Sinica B*.

[33] Kuhla A., Eipel C., Abshagen K., Siebert N., Menger M. D., Vollmar B. (2009). Role of the perforin/granzyme cell death pathway ind-Gal/LPS-induced inflammatory liver injury. *American Journal of Physiology-Gastrointestinal and Liver Physiology*.

[34] Li L., Yin H., Zhao Y. (2018). Protective role of puerarin on LPS/D-Gal induced acute liver injury via restoring autophagy. *American Journal of Translational Research*.

[35] Xu G. M., Zan T., Li H. Y. (2018). Betulin inhibits lipopolysaccharide/D-galactosamine-induced acute liver injury in mice through activating PPAR-*γ*. *Biomedicine & Pharmacotherapy*.

[36] Anderson F. H., Zeng L. C., Rock N. R., Yoshida E. M. (2000). An assessment of the clinical utility of serum alt and ast in chronic hepatitis c. *Hepatology Research*.

[37] Barrera G., Pizzimenti S., Daga M. (2018). Lipid peroxidation-derived aldehydes, 4-hydroxynonenal and malondialdehyde in aging-related disorders. *Antioxidants*.

[38] Li H., Horke S., Förstermann U. (2014). Vascular oxidative stress, nitric oxide and atherosclerosis. *Atherosclerosis*.

[39] Goc Z., Szaroma W., Kapusta E., Dziubek K. (2017). Protective effects of melatonin on the activity of SOD, CAT, GSH-Px and GSH content in organs of mice after administration of SNP. *The Chinese Journal of Physiology*.

[40] Guven A., Guven A., Gulmez M. (2003). The effect of kefir on the activities of GSH-Px, GST, CAT, GSH and LPO levels in carbon tetrachloride-induced mice tissues. *Zoonoses & Public Health*.

[41] Chen Y., Li Q., Kuang Z. P. (2020). Inhibitory effect of flavonoid extract of lotus leaf on alcohol-induced gastric injury by antioxidant capacity in mice. *Journal of Food Quality*.

[42] Kim B. M., Cho B. O., Jang S. I. (2019). Anti-obesity effects of Diospyros lotus leaf extract in mice with high-fat diet-induced obesity. *International Journal of Molecular Medicine*.

[43] Hjertner B., Bengtsson T., Morein B., Paulie S., Fossum C. (2018). A novel adjuvant G3 induces both Th1 and Th2 related immune responses in mice after immunization with a trivalent inactivated split-virion influenza vaccine. *Vaccine*.

[44] Lu T. X., Hartner J., Lim E. J. (2011). Microrna-21 limits in vivo immune response-mediated activation of the IL-12/IFN-gamma pathway, Th1 polarization, and the severity of delayed-type hypersensitivity. *Journal of Immunology*.

[45] Li X., Tang Y., Ma B. (2018). The peptide lycosin-I attenuates TNF-*α*-induced inflammation in human umbilical vein endothelial cells via I*κ*B/NF-*κ*B signaling pathway. *Inflammation Research*.

[46] Revu S., Wu J., Henkel M. (2018). IL-23 and IL-1*β* drive human Th17 cell differentiation and metabolic reprogramming in absence of CD28 costimulation. *Cell Reports*.

[47] Wang X., Yang F., Xu G., Zhong S. (2018). The roles of IL-6, IL-8 and IL-10 gene polymorphisms in gastric cancer: a meta-analysis. *Cytokine*.

[48] Liu S. H., Lu T. H., Su C. C. (2014). Lotus leaf (Nelumbo nucifera) and its active constituents prevent inflammatory responses in macrophages via JNK/NF-*κ*B signaling pathway. *The American Journal of Chinese Medicine*.

[49] Tseng H. C., Tsai P. M., Chou Y. H., Lee Y. C., Lin H. H., Chen J. H. (2019). In VitroandIn VivoProtective effects of flavonoid-enriched lotus seedpod extract on lipopolysaccharide-induced hepatic inflammation. *The American Journal of Chinese Medicine*.

